# Divergent evolution peaks under intermediate population bottlenecks during bacterial experimental evolution

**DOI:** 10.1098/rspb.2016.0749

**Published:** 2016-07-27

**Authors:** Tom Vogwill, Robyn L. Phillips, Danna R. Gifford, R. Craig MacLean

**Affiliations:** Department of Zoology, University of Oxford, South Parks Road, Oxford OX1 3PS, UK

**Keywords:** experimental evolution, population bottlenecks, evolutionary rescue, genome sequencing, parallel evolution

## Abstract

There is growing evidence that parallel molecular evolution is common, but its causes remain poorly understood. Demographic parameters such as population bottlenecks are predicted to be major determinants of parallelism. Here, we test the hypothesis that bottleneck intensity shapes parallel evolution by elucidating the genomic basis of adaptation to antibiotic-supplemented media in hundreds of populations of the bacterium *Pseudomonas fluorescens* Pf0-1. As expected, bottlenecking decreased the rate of phenotypic and molecular adaptation. Surprisingly, bottlenecking had no impact on the likelihood of parallel adaptive molecular evolution at a genome-wide scale. However, bottlenecking had a profound impact on the genes involved in antibiotic resistance. Specifically, under either intense or weak bottlenecking, resistance predominantly evolved by strongly beneficial mutations which provide high levels of antibiotic resistance. In contrast with intermediate bottlenecking regimes, resistance evolved by a greater diversity of genetic mechanisms, significantly reducing the observed levels of parallel genetic evolution. Our results demonstrate that population bottlenecking can be a major predictor of parallel evolution, but precisely how may be more complex than many simple theoretical predictions.

## 1. Introduction

Parallel evolution, where the same beneficial mutations are fixed in independent populations or lineages, has now been documented in a wide range of organisms and in response to a range of selection pressures [1–3]. However, parallelism seems to be particularly common in bacteria, although it is far from universal. For example, some degree of parallel genetic evolution is commonly observed during host specialization in pathogens [4–6] and in endosymbionts [7–9], and parallel evolution in antibiotic resistance genes occurs across highly divergent bacteria [4,5,10,11]. It is unclear, however, what determines the precise level of observed parallel evolution in bacteria. It can partly be explained by bacteria having small compact genomes, orders of magnitude smaller than higher eukaryotes. It is also clear that, in some cases, genetic constraints promote parallel evolution [1,12,13]. For example, there are very few genes in bacterial genomes that can be mutated to produce a high level of resistance to many antibiotics [14–17], and unsurprisingly parallel evolution of resistance by mutations in these genes is common.

In addition to genetic constraints, demographic factors such as population bottlenecks are likely to be a major determinant of the repeatability of adaptation [18–20]. Population bottlenecks are a common and unavoidable aspect of the demography of most organisms, but are practically unavoidable for pathogenic bacteria, due to transmission between hosts as well as strong selection from immune systems and antibiotics. Population bottlenecks can affect adaptation in a variety of ways, but these can be broadly grouped into genetic effects, which can be either stochastic or deterministic, and demographic effects, which are generally more deterministic in that bottlenecks increase mortality. For example, bottlenecks reduce genetic variation by stochastically eliminating rare alleles from populations, and the simplest consequence of bottlenecking is a reduction in the rate of adaptation [19,21]. However, population bottlenecking is also predicted to have important consequences for the genetic mechanisms of adaptation. In large populations that experience weak bottlenecking, independently derived beneficial mutations can compete with each other, which has the potential to eliminate weakly beneficial mutations. The consequence of this effect, known as Hill–Robertson [22] or clonal interference [23], is that adaptation in large populations will be driven by strongly beneficial mutations in a subset of genes that are under strong selection, resulting in a high probability of parallel evolution. This argument is based on classical concepts from population genetics, and is solely based on differences in relative fitness between competing genotypes. Briefly, this argument predicts that increasing the severity of population bottlenecks should decrease the probability of parallel evolution.

In certain circumstances, however, the relationship between bottlenecking and parallelism should not be quite so straightforward, as the increased mortality from bottlenecks can also affect adaptation. If the intensity of bottlenecking is greater than the population growth rate, population size will begin to decline, which will eventually result in extinction unless selection acts to increase the population growth rate. In this scenario, which is often known as evolutionary rescue, the fate of beneficial mutations depends on how they alter absolute fitness as opposed to relative fitness [24,25]. For example, weakly beneficial mutations, which only lead to small increases in fitness, may not be able to fix in response to stringent bottlenecking [26,27]. This is because these mutations will have a net reproductive rate that is effectively smaller than zero after the additional mortality associated with bottlenecking is taken into account. Therefore, despite increasing relative fitness, they would not increase absolute fitness. Briefly, strong bottlenecking is expected to also lead to the disproportionate loss of weakly beneficial mutations [26,27], and, therefore, bias selection to just a subset of genes that have large phenotypic effects, again leading to high levels of parallel evolution.

Taking these two arguments together, it could be predicted that either intermediate bottlenecking should lead to the lowest levels of parallel evolution, or alternatively that bottlenecking does not affect the probability of parallel evolution. In this paper, we test the role of population bottlenecking on the rate and mechanisms of adaptation using an experimental model system. We propagated hundreds of populations of the bacterium *Pseudomonas fluorescens Pf0-1* in a standard laboratory culture medium supplemented with the antibiotic rifampicin. We manipulated the strength of daily population bottlenecking over 1 order of magnitude (200-fold to 2 000-fold reduction in population density) by changing the fraction of each population that was transferred to a fresh culture medium on a daily basis. Crucially, the combination of a potent dose of antibiotic and population bottlenecking used in our experiment ensured that populations from all bottleneck treatments could only persist until the end of the experiment by evolving an increased growth rate. Thus, our experiment challenged bacterial populations with ‘evolutionary rescue’.

Previous experiments that have investigated the impact of population bottlenecking on parallel evolution have focused on testing for parallelism at a phenotypic level [28–30], by measuring divergence between populations in phenotypic traits that are closely linked to fitness. Tests on the role of bottlenecks, or even more generally, population size, are rare for molecular evolution (but see [31]). We, therefore, tested our hypothesis using both phenotypic assays but also whole-genome sequencing, which would allow us to take a hierarchical approach to testing parallel evolution [2,32,33].

## 2. Material and methods

### (a) Strains, culture conditions, and antibiotic

*Pseudomonas fluorescens Pf0-1* was obtained from Gail Preston (Department of Plant Sciences, University of Oxford, UK) in January 2012. Prior to experimentation, it was stored at −80°C in 25% glycerol. All culturing was performed in King's B (KB) media, at 30°C with constant shaking at 250 r.p.m. Rifampicin is an inhibitor of RNA polymerase and was stored according to the manufacturer's instructions.

### (b) Selection experiment

We used a sublethal dose of rifampicin as the main selection pressure. The minimum inhibitory concentration of rifampicin had been previously determined for *Pf0-1* under our experimental conditions (8 µg ml^−1^), and we used 80% of this concentration in our experiment (6.4 µg ml^−1^). This limited the ancestral growth rate to 10% of its maximum, and effectively created a declining population, akin to evolutionary rescue conditions.

We used three different bottleneck sizes to manipulate population size. Specifically, we diluted selection lines 1/200, 1/600, or 1/2 000 into 200 µl of fresh KB media containing rifampicin on a daily basis, henceforth referred to as weak, intermediate, and strong bottleneck treatments, respectively. To begin the experiment, a single colony of *Pf0-1* was isolated by streaking on agar. It was inoculated in 1 ml of KB media, grown overnight at 30°C, and then used to found 96 replicates of the weak bottleneck treatment, 96 replicates of the intermediate bottleneck treatment, and 192 replicates of the strong bottleneck treatment. This is approximately 2 × 10^6^, 6 × 10^5^, and 2 × 10^5^ cells, respectively, transferred at the start of the experiment. However, after the initial transfer, the number of cells transferred will decrease with each transfer, unless populations adapt, in which case it may be greater than these values.

As variable bottlenecks also vary the maximum number of generations per day (if all populations were to return to the same density after each transfer), we ran the selection experiment for 14, 12, and 10 days, respectively. This results in approximately 110 total generations, assuming ancestral growth. During the experiment, every 2 days samples of all populations were transferred to 25% glycerol and stored at −80°C.

### (c) Sequencing

Following the selection experiment, a single colony was isolated for genomic sequencing from 34 randomly chosen weak bottleneck populations, 33 randomly chosen intermediate bottleneck populations, and all 26 surviving strongly bottlenecked populations. Although sequencing a single clone ignores any within-population diversity, given current coverage levels/sequencing technology it provides the most convenient way to measure between-population parallelisms. Genomic DNA was extracted from these 93 clones using the Promega Genomic Wizard kits, and the protocol was otherwise performed as per the manufacturer's instructions. DNA was then quantified using the Quantifluor dsDNA system from Promega. Sequencing was conducted by the Wellcome Trust Centre for Human Genetics using HiSeq2000 and 100 bp paired-end reads. We also sequenced three clones of the ancestor used to initiate the experiment, to detect differences from the published reference.

### (d) Bioinformatics

We analysed the sequencing data using the method first described in [34] (see also the electronic supplementary material). Briefly, quality checked reads were aligned to the *Pf0-1* reference sequence (NC_007492.2) using Burrows–Wheeler Aligner (BWA). We called variants (e.g. single nucleotide polymorphisms (SNPs), large and small indels, copy number variants, inversions, translocations) using multiple tools: GATK Unified Genotyper [35], samtools mpileup [36], BreakDancer [37], Pindel [38], and Control-FREEC [39]. Variants were annotated using SnpEff [40]. Information about gene function was obtained from the Pseudomonas Genome Database [41].

### (e) Fitness assays

We used growth rate in the presence of rifampicin as a proxy for fitness in the evolved clones. Specifically, we measured the rate of exponential growth of each clone grown in the presence of the experimental dose of rifampicin. For each assay, each clone was grown overnight in KB media, diluted 1 000-fold in KB media containing rifampicin and grown overnight at 30°C with constant shaking at 250 r.p.m. OD600 readings were taken every 20 min using a BioTek synergy plate reader (Winooski, VT). Each assay was replicated four times under these conditions. Assays were performed in blocks, with each clone assayed in at least three different blocks, with two replicates per block. Blocks were standardized by subtracting the mean growth rate of six ancestral controls included within each block. We defined exponential growth rate as the maximum rate of growth over six consecutive readings. Subsets of assays were repeated using dilutions of 200×, 600×, and 2 000× during inoculation to mimic the experimental transfer sizes. The inoculum levels of these assays did not affect the maximum rate of growth, at least at the bottleneck sizes used here.

## 3. Results

### (a) Bottleneck intensity and phenotypic evolution

**Figure 1.**
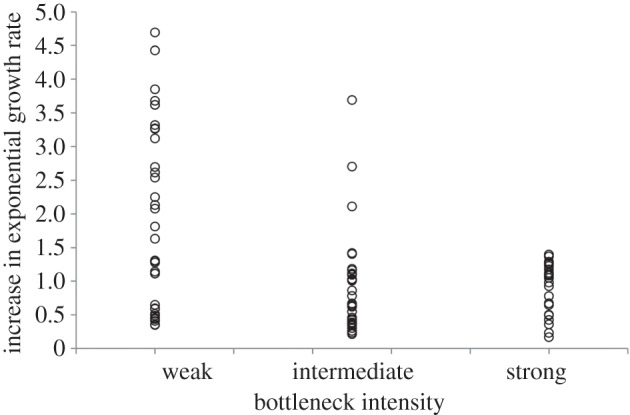
The impact of population bottlenecking on adaptation. Each symbol indicates the fitness of independently evolved clones, as measured by growth rate in the presence of rifampicin relative to the ancestor growing in the presence of rifampicin.

Population genetics theory predicts that population bottlenecking should constrain adaptation by reducing genetic diversity and increasing the rate of population decline, which we tested in two ways. First, we assayed the proportion of populations which had gone extinct during the selection experiment, by plating samples of all populations from the end of the experiment onto agar plates lacking rifampicin, the dominant selective pressure in the experiment. These plates would, therefore, be permissive to any non-adapted cells still present in any populations at the end of the experiment. As predicted by theory, the strong bottleneck treatment led to a high probability of extinction (86%) while more relaxed bottlenecks resulted in far less extinction (48% and 8%, respectively). Secondly, we assayed the fitness of clones from a subset of surviving populations from each treatment, by measuring the absolute fitness (i.e. growth rate) of independently evolved clones sampled at the end of the experiment (figure 1). Specifically, we assayed a single clone from each of 34 weakly bottlenecked populations, from each of 33 medium bottlenecked populations, and from all 26 surviving strongly bottlenecked populations. As expected, the clones from the weak bottleneck treatment show significantly higher fitness than either of the other treatments (Bonferroni-corrected *t*-tests: weak versus intermediate: *t* = 3.78, d.f. = 65, *p* < 0.001; weak versus strong: *t* = 3.82, d.f. = 58, *p* < 0.001; strong versus intermediate: *t* = 0.16, d.f. = 57, *p* = 0.873). Therefore, even when adaptation was able to prevent population extinction, population bottlenecking constrained the efficacy of natural selection.

Evolutionary theory also predicts that bottlenecking should lead to increased divergence between populations. To test this hypothesis, we estimated the variance component independently for each treatment, fitting a model taking into account both variances between clones as well as experimental error. We found that in contrast with theoretical expectation, variance between clones decreased with increasing bottleneck intensity (variance components; weak = 0.864, intermediate = 0.208, strong = 0.017; pairwise *F*-tests on variance: weak versus intermediate: *F*_33,32_ = 4.15, *p* < 0.001; weak versus strong: *F*_33,25_ = 50.8, *p* < 0.001; intermediate versus strong: *F*_32,25_ = 12.2, *p* < 0.001). Therefore, at the phenotypic level, evolution was most parallel with the strongest bottlenecks.

### (b) Bottlenecking and genome-wide divergent molecular evolution

To determine the molecular basis of adaptation, we sequenced the genome of each of the 93 clones used for the fitness assays. We also sequenced three clones from the ancestral stock, to identify differences between the starting point of our experiment and the published reference sequence. In total, we identified 259 mutations across these clones (mean 2.78 mutations per clone, range 1–6), spread across 91 loci including 66 genes. Specifically, we identified 174 intragenic non-synonymous SNPs, 7 synonymous intragenic SNPs, 51 intragenic indels, 22 intergenic mutations, and 5 large deletions (greater than 50 bp). Parallel evolution was reasonably common, with 19 genes mutated in at least two independent clones, accounting for 71.4% of all detected mutations. Given the high levels of repeated gene use, and low proportion of synonymous mutations, it is reasonable to assume that the majority of detected mutations are at least weakly beneficial.

**Figure 2.**
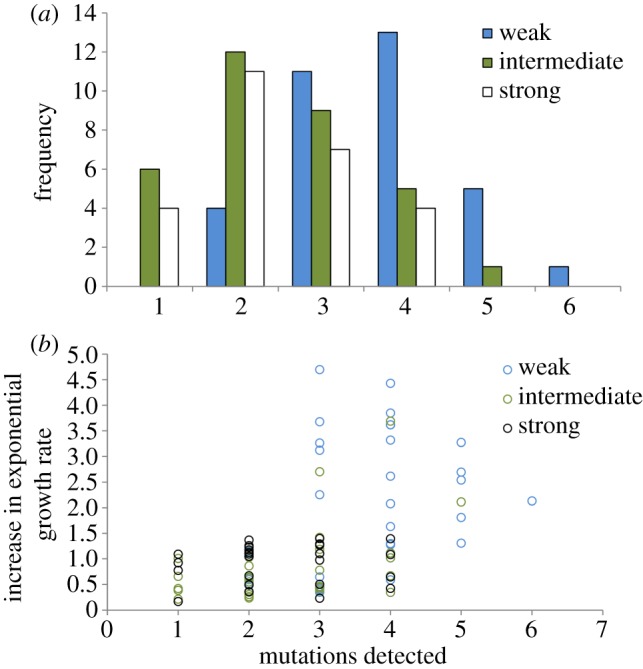
The impact of bottlenecking on the rate of molecular adaptation. Panel (*a*) shows the number of mutations detected per clone as a function of bottlenecking intensity (weak bottleneck: mean (±s.e.) = 3.65 ± 0.17; intermediate: mean (±s.e.) = 2.48 ± 0.19; strong: mean (±s.e.) = 2.42 ± 0.19). Plotted points in (*b*) show the fitness of independently evolved clones as a function of the number of mutations acquired during the experiment. Fitness was measured as relative growth rate in the presence of rifampicin, and fitness increases with mutation number.

Interestingly, we found that the number of mutations per clone decreased with increasing intensity of bottlenecking (generalized linear model with Poisson's distribution and log-linked function: Wald *χ*^2^ = 10.2, d.f.= 2, *p* < 0.01; figure 2*a*). Specifically, we detected significantly more mutations per clone in the weak bottleneck treatment than either of the other treatments (Bonferroni-corrected *t*-tests: weak versus intermediate: *t* = 4.65, d.f. = 65, *p* < 0.001; weak versus strong: *t* = 4.86, d.f. = 58, *p* < 0.001; strong versus intermediate: *t* = 0.23, d.f. = 57, *p* = 0.82). Given that fitness evolves most rapidly in weakly bottlenecked populations, the link between population bottlenecking and the rate of molecular evolution provides further evidence to support the idea that the majority of detected mutations were beneficial. Indeed, we find that the number of mutations per clone significantly correlates with fitness, even after correcting for the effect of bottleneck size (general linear model on fitness with bottleneck intensity as a fixed factor and mutations-fixed as a covariate; bottleneck: *F*_2,89_ = 4.7, *p* < 0.05; mutations: *F*_1,89_ = 7.89, *p* < 0.01).

**Figure 3.**
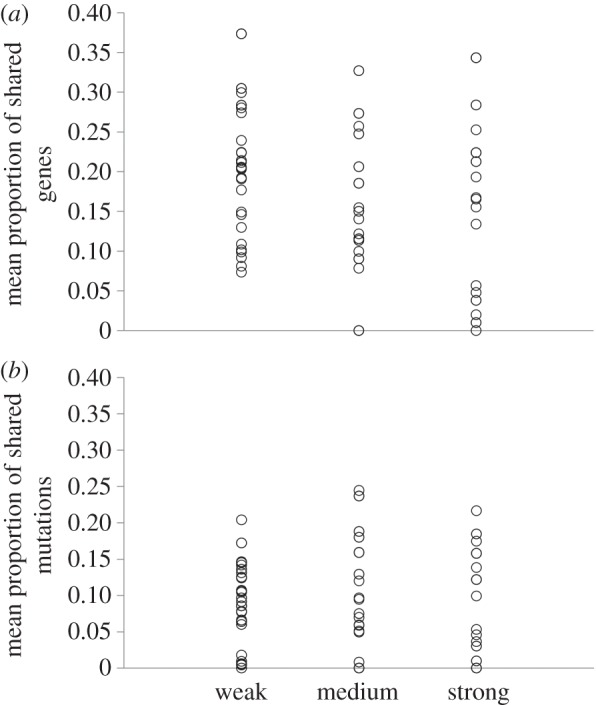
The impact of population bottlenecking on parallel evolution. Parallelism was measured as the mean proportion of shared mutations between pairs of clones that evolved under the same bottlenecking treatment using the Jaccard index. (*a*) Parallel evolution at the level of genes and (*b*) parallelism at the level of individual SNPs.

To test the hypothesis that population bottlenecking alters the probability of parallel evolution, we first calculated a distance matrix using Jaccard's index [42]. This index is commonly used to assay parallel evolution and measures the proportion of genetic changes in common between a pair of clones. When calculated in a pairwise manner for all clones within the same group, it provides a measure of mean within group-parallel evolution. Interestingly, bottleneck intensity did not affect the mean proportion of shared mutated genes (permutational analysis of multivariate homogeneity of group dispersion [43]: *F*_2,90_ = 0.988, *p* = 0.386; figure 3*a*) or shared SNPs (permutational analysis of multivariate homogeneity of group dispersion: *F*_2,90_ = 0.091, *p* = 0.919; figure 3*b*). However, even if the level of parallel evolution is the same in all groups, it does not mean all groups are fixing the same mutations. Using permutational multivariate analysis [44] of the Jaccard distance matrix (equivalent to a one-way ANOVA on univariate data) reveals that is indeed the case for both genes (*F*_2,90_ = 3.67, *p* < 0.001) and SNPs (*F*_2,90_ = 1.86, *p* < 0.01). In other words, the mean number of shared mutations is higher within groups than between them.

### (c) Evolution of major genes

To identify which genes were more likely to be mutated in particular bottleneck treatments, we decided to focus our analysis on genes which were likely to have a large fitness effect. The dose of rifampicin used in our experiment reduced the growth rate of the ancestral clone by 90%, implying that antibiotic resistance mutations are a reasonable candidate to be strongly beneficial. In support of this idea, the two most commonly mutated genes (representing more than a third of total mutations) are both known to confer antibiotic resistance. The most commonly mutated gene was the beta-subunit of RNA polymerase (*rpoB*; 64 mutations across 59 clones), which is unsurprising because *rpoB* mutations are the major mechanism of clinical rifampicin resistance [45]. The second most common target of selection was *cpxA* (Pfl01_1481) (39 mutations across 39 clones), a regulator of the cell envelope stress response [46]. *cpxA* regulates several efflux pumps known to be associated with antibiotic resistance [46–49], although not previously involved in resistance to rifampicin. Only 8 of the 93 clones lacked mutations in either of these genes, suggesting these two genes are the two major mechanisms of resistance.

**Figure 4.**
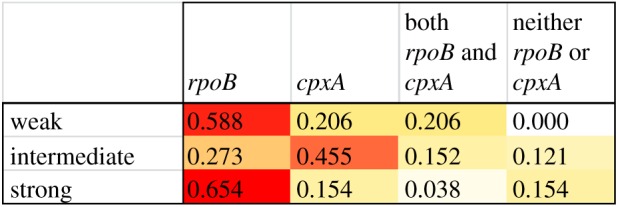
The impact of bottlenecking on resistance mechanisms. This figure is a heat map showing the frequency of mutations in *rpoB* and *cpxA* across bottlenecking treatments. *rpoB* is more common with either weak or strong bottlenecks, while *cpxA* is most common at intermediate bottlenecks. The intermediate treatment shows the least bias to any one mechanism, and consequently the highest diversity of mechanisms.

**Figure 5.**
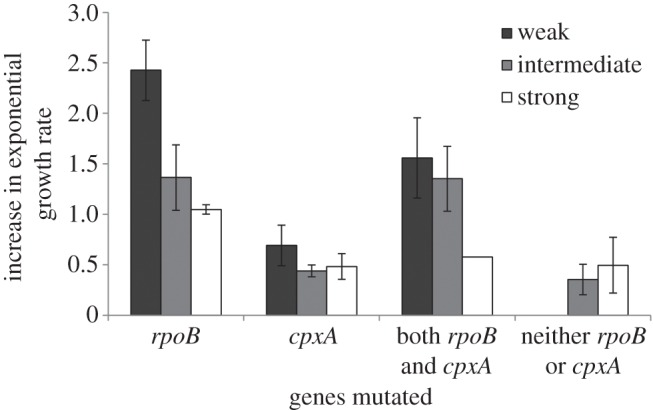
Fitness effects of alternative resistance mechanisms. Bars show the mean (±s.e.m) fitness of clones according to resistance mechanism and bottlenecking intensity.

Intriguingly, bottleneck intensity had a significant impact on the the molecular mechanisms of antibiotic resistance (*χ*^2^-test on the proportion of clones with mutations in *rpoB* and *cpxA*: *χ*^2^ = 18.75, *p* < 0.005; figure 4). Under both weak and strong bottlenecking, *rpoB* mutations predominated, while by contrast, *cpxA* mutations were more common under intermediate bottlenecking. However, in general, the intermediate bottleneck treatment showed less bias towards any one particular mechanism, and consequently, showed the highest diversity of resistance mechanisms and the lowest probability of parallel evolution (Simpson's index of diversity of resistance mechanisms: strong bottlenecks = 0.524, intermediate bottlenecks = 0.681, weak bottlenecks = 0.569). To explain why the diversity of mechanisms was highest at intermediate bottlenecks, we reanalysed the fitness data with respect to resistance mechanisms. As shown, clones with mutations in *rpoB* are significantly fitter than clones with mutations in *cpxA* (one-way ANOVA on ‘clones with mutations in *rpoB* but not *cpxA*’ versus ‘clones with mutations in *cpxA* but not *rpoB*’: *F*_1,70_ = 21.90, *p* < 0.001; figure 5)*.* This is still true if the analysis is limited to clones possessing only a single mutation in *rpoB* (mean fitness = 0.798 ± 0.078, *n* = 3) or *cpxA* (mean fitness = 0.499 ± 0.104, *n* = 4) and no other mutations anywhere else in their genome (one-way ANOVA on clones with only a mutation in *rpoB* versus clones with only a mutation in *cpxA*: *F*_1,5_ = 6.73, *p* < 0.05). Therefore, both strong and weak bottlenecks lead to a bias towards the more strongly beneficial mutations, and consequently, divergence is not greatest at the strongest bottleneck. However, for intermediate bottlenecks, there is less bias towards strongly beneficial mutations, and consequently, greater diversity.

### (d) Epistasis between major genes

From figure 5, it can be seen that clones possessing mutations in both *rpoB* and *cpxA* would appear to have intermediate fitness compared with clones only possessing mutations in one of these two genes. However, the fitness of clones with mutations in *rpoB* does not significantly differ if the clone also has a mutation in *cpxA* (independent sample *t*-test: clones with *rpoB* versus clones with both *rpoB* and *cpxA*: *t* = 0.867, d.f. = 57, *p* = 0.365). As both *rpoB* and *cpxA* mutations increase growth rate, this demonstrates negative epistatic fitness effects for these two genes. In other words, the fitness benefit of having mutations in both genes is less than expected from the fitness effects of mutations in either of the two genes alone.

### (e) Evolution within major genes

**Figure 6.**
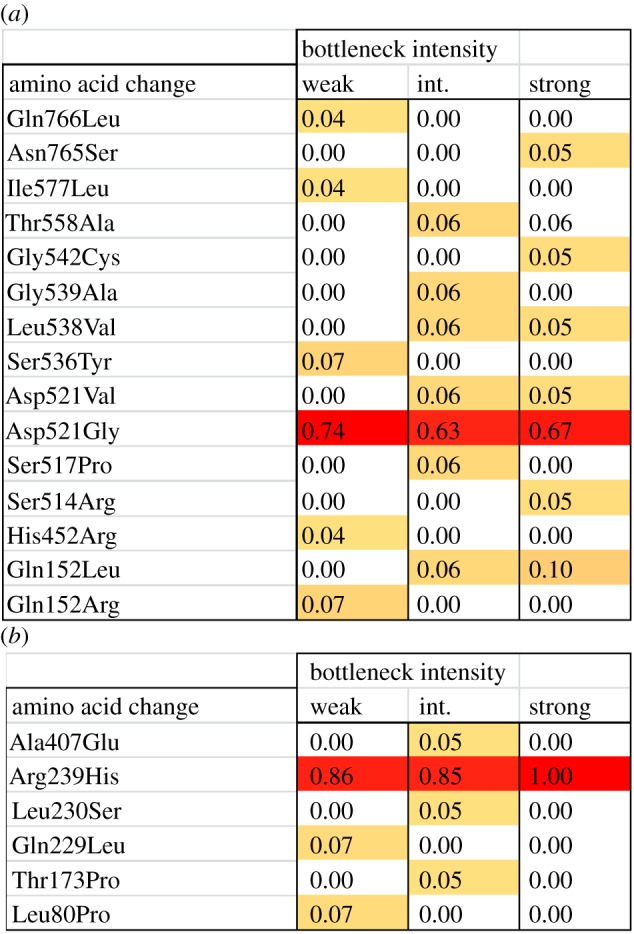
The impact of bottlenecking on evolution within resistance genes. This figure shows a heat map of the frequency of SNPs within (*a*) *rpoB* and (*b*) *cpxA* across bottlenecking treatments.

Given that most of the dynamics of fitness were being driven by mutations in just two genes, we tested whether bottleneck intensity was affecting which nucleotides were being selected within these genes. Previous work has shown that different SNPs in *rpoB* can have different effects on bacterial fitness by altering both the level of rifampicin resistance, as well as competitive ability and growth rate. Given this diversity of fitness effects, as well as the large number of possible mutations in *rpoB*, different spectra of *rpoB* substitutions might be expected to evolve in response to varying bottleneck intensity. However, we find no evidence that this was occurring, suggesting selection was weaker within genes than between genes (*χ*^2^-test on distribution of *rpoB* SNPs: *χ*^2^ = 30.40, *p* = 0.2; figure 6*a*). A similar argument can intuitively be expected to apply to mutations within *cpxA*, the second most common target of selection. However, again we find no evidence of selection favouring differing SNPs within different bottleneck treatments (*χ*^2^-test on the distribution of *cpxA* SNPs: *χ*^2^ = 6.53, *p* = 0.3; figure 6*b*).

## 4. Discussion

Bottlenecks are a common and unavoidable aspect of the demography of most organisms. In this experiment, we studied the phenotypic and genetic consequences of population bottlenecking during adaptation. In our experiment, bottlenecking had a profound impact on the likelihood of adaptation, on the rate of fitness evolution, and on the rate of substitution of mutations. These effects are simple to understand using conventional population genetics reasoning: bottlenecking reduces the effective population size, resulting in a greater rate of loss of beneficial mutations to genetic drift.

However, the impact of population bottlenecking on patterns of molecular evolution is more subtle. Our experimental design imposed strong selection for antibiotic resistance and *P. fluorescens* evolved resistance predominantly using two different genes. One of these appears to be strongly beneficial and is associated with relatively high fitness (*rpoB*), while the other is only weakly beneficial and results in more modest fitness gains (*cpxA*)*. rpoB* mutations prevent rifampicin from binding to its target domain [50] and this is associated with elevated rifampicin resistance and decreased competitive ability, due to the pleiotropic effects of resistance mutations [51,52]. The role of *cpxA* mutations in rifampicin resistance is less well understood, but *cpxA* regulates many efflux pumps known to confer antibiotic resistance [47–49]. Under weak bottlenecking, the higher population size meant an increased probability of two beneficial mutations being present in each population, and competition between independent beneficial mutations therefore favoured *rpoB* over *cpxA*. By contrast, under stronger bottlenecking the higher absolute fitness of *rpoB* mutations reduced the likelihood of stochastic loss at each bottleneck event, again resulting in a disproportionate loss of weakly beneficial *cpxA* alleles. Collectively, these biases resulted in a high likelihood of parallel evolution under either intense or weak bottlenecking. Interestingly, this is an effect that can only be understood by considering the impact of beneficial mutations on absolute fitness, and not relative fitness alone, a distinction often emphasized by evolutionary rescue theory (reviewed in [24]).

However, more broadly, we found the level of parallel molecular evolution at a genome-wide scale was insensitive to population bottlenecking. A major contributor to this is the sheer diversity of evolution at a genome-wide scale, even under the relatively simple laboratory conditions we employed. Although we found considerable evidence for parallel evolution, nearly 30% of all mutations were in genes only mutated in a single clone. Coupled with this, much of the variation in fitness can be attributed to a small subset of genes directly involved in adaptation to rifampicin. Although the dynamics of these major genes were significantly affected by bottlenecking, statistically this affect is hidden by the diffuse nature of evolution across the rest of the genome. In other words, our data suggest that most mutations were only weakly beneficial at best, and consequently, their dynamics were not influenced by the intensities of bottlenecking imposed by our experiment.

Similarly, we also failed to detect any effect of population bottlenecking on the frequency of particular SNPs within major genes. This is likely because in most cases there will be greater variation in the fitness effects of mutations in different genes rather than between different mutations within the same gene. Consequently, selection will be more evident between genes than within them. This is not to say that there cannot be considerable diversity in the phenotypic effects of different nucleotide substitutions within a particular gene (e.g. [51,53,54]). However, these within-gene differences are likely to be most important when adaptation is only possible, or at least most likely, through a single gene, such as the strong selection imposed by clinical doses of antibiotics.

Adaptation is often predicted to be most repeatable at large population sizes, because clonal interference is most prevalent in large populations. Indeed, this is supported by several publications which measured repeatability at the phenotypic level [28–30]. Parts of our results are in agreement with this line of reasoning, such as the high levels of parallel evolution in the weak bottlenecking treatment. However, in our experiment, extinction was just as effective as clonal interference in limiting which mutations could fix, and consequently, we did find limited evidence of repeatability increasing with population size. Therefore, our results suggest caution should be taken in assuming a large population size will always lead to the most repeatable adaptation, particularly in contexts where clonal interference is unlikely to be the only factor influencing adaptation.

In this paper, we only used a single relatively low concentration of rifampicin. If a stronger concentration had been used, it is likely that parallel evolution would have been more common across all bottleneck treatments, as mutations in few genes can result in high-level antibiotic resistance [10]. This is particularly true for rifampicin, as almost all clinical rifampicin resistance mutations are within *rpoB* [45]. Similarly, we only used three bottleneck intensities, which only capture a fraction of the bottleneck sizes which are likely to occur in clinical pathogens. Hopefully, future work will measure the intensity of bottlenecking experienced by bacterial pathogens *in vivo* due to transmission and host immune responses, and thereby provide a guide for future *in vitro* investigation.

Parallel evolution is common in bacteria both in natural (e.g. [4–6]) and laboratory environments (e.g. [11,55]). Given the difficulties associated with applying many classical tests for positive selection to bacterial populations [56], it has been suggested that parallel evolution should be used to test for positive selection [57]. Our results suggest both optimism and caution towards this approach. Theoretical reasoning and previous experiments suggest that this may be a dangerous approach to use, as population demography might play an important role in shaping the likelihood of parallel evolution [28–30]. However, our results suggest that genome-wide patterns of parallelism may be relatively independent from population bottlenecking, which is likely to be a key feature of the demography of many bacteria, especially bacterial pathogens. An important caveat is that population bottlenecking might have a strong effect on patterns of parallel evolution in genes that are likely to be under strong selection, such as antibiotic resistance genes. More generally, the causes of parallel evolution in bacteria remain unclear. Our data suggest that competition between beneficial mutations could be a predominant factor, but equally our data suggest that high levels of parallel molecular evolution can still happen in the absence of this competition.

## Supplementary Material

Sequencing supplement

## Data accessibility

Experimental data has been deposited into Dryad (doi:10.5061/dryad.640j4). Sequences have been deposited into the European Nucleotide Archive with the study reference PRJEB14763, available at the URL: http://www.ebi.ac.uk/ena/data/view/PRJEB14763.
