# Supplementary material for: Divergent evolution peaks under intermediate population bottlenecks during bacterial experimental evolution
Source: Proc Biol Sci. 2016 Jul 27;283(1835):20160749. doi: 10.1098/rspb.2016.0749 (PMC4971204; doi:10.1098/rspb.2016.0749)
Supplement: Sequencing supplement [file rspb20160749supp1.pdf]

Bioinformatics supplementary methods

Analysis of whole genomes

Analysis of whole genomes was performed as described in San Millan et al. (2014). Whole genome sequencing services were provided by the Wellcome Trust Centre for Human Genetics (Oxford, UK) using the Illumina HiSeq2500 platform with 100 bp paired-end reads. Initial read filtering was done using NIH QC Toolkit (Patel and Jain, 2012). 5' or 3' ends were trimmed if the Phred quality score was less than 20. Reads were discarded: 1) if they were shorter than 50 bp after trimming, 2) if more than 2% of bases were ambiguous, or 3) if more than 20% of bases had a Phred score < 20.

We mapped the filtered reads to the *P. fluorescens Pf0-1* reference genome (NC_007492.2) using BWA. Mapped reads were processed to increase the quality of the variant calling: 1) reads with multiple best hits were discarded; 2) duplicated reads were discarded using MarkDuplicates from the Picard package (http://picard.sourceforge.net); 3) reads around indels were locally realigned using RealignerTargetCreator and IndelRealigner from the GATK package to correct for misalignment; and 4) mate pairs were sorted using FixMateInformation in the Picard package.

Variant calling was performed with GATK's Unified Genotyper (De Pristo et al., 2011) and Samtools's mpileup (Li et al., 2009). VCFtools (vcf-annotate, Danecek et al., 2011) and GATK toolkit (VariantFiltration, DePristo et al., 2011) were used to filter the raw variants for strand bias, end distance bias, base quality bias, SNPs around gaps, low coverage and erroneously high coverage. All variants called were merged using GATK's CombineVariants (keeping any unfiltered variants). High quality variants not removed by filtering were annotated using SnpEff (Cingolani et al., 2012).

We detected structural variants using three approaches: 1) BreakDancer (Chen et al., 2009) was used to predict deletions, insertions, inversions, and translocations using deviations in the separation or orientation of mapped read pairs; 2) Pindel (Ye et al., 2009) was used to infer deletions, short insertions, long insertions, inversions, tandem duplications, and breakpoints using a split-read approach (the output of BreakDancer was also fed to Pindel to improve its output); 3) Control-FREEC (Boeva et

al., 2011) was used to detect copy number variants (CNVs). Control-FREEC finds CNVs using depth-of-coverage (normalized by GC-content). Regions of low mappability were excluded by supplying Control-FREEC with mappability tracks generated by gem-mappability (GEM library, Marco-Sola et al., 2012).

References

Chen, K., J. W. Wallis, M. D. McLellan, D. E. Larson, J. M. Kalicki, C. S. Pohl,S. D. McGrath, M. C. Wendl, Q. Zhang, D. P. Locke, et al. 2009. BreakDancer: an algorithm for high-resolution mapping of genomic structural variation. Nature Methods 6:677–681.

Cingolani, P., A. Platts, M. Coon, T. Nguyen, L. Wang, S. J. Land, X. Lu, D. M. Ruden, et al. 2012. A program for annotating and predicting the effects of single nucleotide polymorphisms, SnpEff: SNPs in the genome of *Drosophila melanogaster* strain w1118; iso-2; iso-3. Fly 6:80–92.

Danecek, P., A. Auton, G. Abecasis, C. A. Albers, E. Banks, M. A. DePristo, R. E. Handsaker, G. Lunter, G. T. Marth, S. T. Sherry, et al. 2011. The variant call format and VCFtools. Bioinformatics 27:2156–2158.

DePristo, M. A., E. Banks, R. Poplin, K. V. Garimella, J. R. Maguire, C. Hartl,A. A. Philippakis, G. del Angel, M. A. Rivas, M. Hanna, et al. 2011. A frame-work for variation discovery and genotyping using next-generation DNA sequencing data. Nature Genetics 43:491–498.

Li, H., B. Handsaker, A. Wysoker, T. Fennell, J. Ruan, N. Homer, G. Marth,G. Abecasis, R. Durbin, et al. 2009. The sequence alignment/map format and SAMtools. Bioinformatics 25:2078–2079.

Marco-Sola, S., M. Sammeth, R. Guigó, and P. Ribeca. 2012. The GEM map-per: fast, accurate and versatile alignment by filtration. Nature Methods 9:1185–1188.

Patel, R. K., and M. Jain. 2012. NGS QC Toolkit: a toolkit for quality control of next generation sequencing data. PLoS One 7:e30619.

Ye, K., M. H. Schulz, Q. Long, R. Apweiler, and Z. Ning. 2009. Pindel: a pattern growth approach to detect break points of large deletions and medium sized insertions from paired-end short reads. Bioinformatics 25:2865–2871.
